# Frequency and distribution of *FCN2* and *FCN3* functional variants among *MBL2* genotypes

**DOI:** 10.1007/s00251-016-0903-4

**Published:** 2016-01-21

**Authors:** Helga Bjarnadottir, Margret Arnardottir, Bjorn Runar Ludviksson

**Affiliations:** Department of Immunology, Landspitali—The National University Hospital of Iceland, Hringbraut (Building 14 at Eiriksgata), 101 Reykjavik, Iceland; Faculty of Medicine, University of Iceland, Reykjavik, Iceland

**Keywords:** Lectin pathway, Complement, Mannan-binding lectin, Ficolin-2, Ficolin-3, MASP-2, *MBL2* genotypes, *FCN2* + *6424*, *FCN3* + *1637delC*, Redundancy

## Abstract

The six types of pattern recognition molecules (PRMs) that initiate complement via the lectin pathway (LP) comprise collectins and ficolins. The importance of having various PRMs to initiate the LP is currently unclear. Mannan-binding lectin (MBL) is a collectin member of the LP PRMs. MBL deficiency is common with mild clinical consequence. Thus, the lack of MBL may be compensated for by the other PRMs. We hypothesized that variants *FCN2* + *6424* and *FCN3* + *1637delC* that cause gene-dose-dependent reduction in ficolin-2 and ficolin-3 levels, respectively, may be rare in MBL-deficient individuals due to the importance of compensation within the LP. The aim of this study was to investigate the distribution and frequency of these variants among *MBL2* genotypes in healthy subjects. The allele frequency of *FCN2* + *6424* and *FCN3* + *1637delC* was 0.099 and 0.015, respectively, in the cohort (*n* = 498). The frequency of *FCN2* + *6424* tended to be lower among MBL-deficient subjects (*n* = 53) than among MBL-sufficient subjects (*n* = 445) (0.047 versus 0.106, *P* = 0.057). In addition, individuals who were homozygous for *FCN2* + *6424* were sufficient MBL producers. The frequency of *FCN3* + *1637delC* did not differ between the groups. The frequency of *FCN2* + *6424* was similar in *FCN3* + *1637delC* carriers (*n* = 15) versus wild type (*n* = 498). Furthermore, subjects that were heterozygote carriers of both *FCN2* + *6424* and *FCN3* + *1637delC* were sufficient MBL producers. In conclusion, *FCN2* + *6424* carriers with MBL deficiency tend to be rare among healthy individuals. MBL-deficient individuals with additional LP PRM defects may be at risk to morbidity.

## Introduction

The lectin pathway (LP) is the most recently discovered of the three complement activation pathways. It is initiated by one of the six pattern recognition molecules (PRMs), either mannan-binding lectin (MBL), collectin kidney-1 (CL-K1), collectin liver-1 (CL-L1), ficolin-1 (M-ficolin), ficolin-2 (L-ficolin) or ficolin-3 (H-ficolin) (Henriksen et al. 2013; Kjaer et al. 2013). The PRMs bind specific molecular patterns on pathogens, apoptotic cells and cellular debris as they circulate in serum complexed with three serine proteases (MASP-1–3) and two non-enzymatic proteins with regulatory functions: MAP1 and sMAP (Dahl et al. 2001; Degn et al. 2009; Sato et al. 1994; Skjoedt et al. 2011; Stover et al. 1999; Takahashi et al. 1999; Thiel et al. 1997). Upon binding, MASP-1 activates MASP-2 which subsequently cleaves complement factors C2 and C4 resulting in the activation of the complement cascade with the generation of inflammatory mediators (C3a and C5a), assembly of the membrane attack complex (MAC) and opsonisation (Degn et al. 2012; Héja et al. 2012; Takahashi et al. 2008).

The collectins (MBL, CL-K1 and CL-L1) and ficolins are similar in structure (Kjaer et al. 2013). They are oligomers and share amino terminal collagen-like stalk that interacts with the MASPs. They differ at the carboxyl terminal where the ficolins contain fibrinogen-like recognition domain and the collectins contain C-type carbohydrate recognition domain. Despite having a common structure, ficolin-2 and ficolin-3 are only 48 % identical by sequence (Sugimoto et al. 1998). Both ficolin-2 and ficolin-3 have been shown to bind dying host cells and mediate their clearance through complement activation via the LP (Honoré et al. 2007; Jensen et al. 2007; Kuraya et al. 2005). Ficolin-2 binds various clinically relevant pathogens including those affecting the respiratory system, whereas ficolin-3 does not (Hummelshøj et al. 2012; Krarup et al. 2005; Pan et al. 2012). However, recently, it was shown that ficolin-3 binds enteropathogenic and enteropathoaggregative *Escherichia coli* (Sahagún-Ruiz et al. 2015). Furthermore, the first evidence of antimicrobial activity of ficolin-3 was recently reported against the enteric commensal and opportunistic gut bacteria *Hafnia alvei* (Michalski et al. 2015a). Thus, there appears to be a link between ficolin-3 and the gut. Notably, ficolin-3 is resistant to collagenases (whereas the other ficolins and collagens are not) and this may reflect the site of its antimicrobial activity, i.e. in the gastrointestinal tract (Hummelshoj et al. 2008).

The concentration of MBL in serum ranges from <10–12,200 ng/ml in adults, with a median of 1340 ng/ml (Sallenbach et al. 2011). This large range span can be explained by combinations of functional single nuclear polymorphisms (SNPs) in exon 1 and in the promoter of the *MBL2* gene (Garred et al. 2003). The SNPs in exon 1 have been named variant allele *D* (the codon 52 allele or p.Arg52Cys), *B* (the codon 54 allele or p.Gly54Asp) and *C* (the codon 57 allele or p.Gly57Glu). Collectively, they are referred to as the *O* allele, whereas the normal wild-type allele is referred to as the *A* allele. The *O* allele gives rise to dysfunctional forms of MBL that are unable to bind to their ligands (Lipscombe et al. 1995; Madsen et al. 1994; Sumiya et al. 1991). The SNPs in the *MBL2* promoter region are located at position −50 (H/L) and −221 (X/Y) and at position +4 (P/W) in the 5′-untranslated portion of exon 1 (Madsen et al. 1995; Madsen et al. 1998; Steffensen et al. 2000). The variant at position −221 (*X* allele) has the strongest downregulating effect on MBL expression. A combination of the SNPs in exon 1 and *MBL2* promoter determines various *MBL2* genotypes. The *XA*/*O* and *O*/*O* genotypes are generally referred to as MBL-deficient genotypes as they give rise to undectable MBL levels (<10 ng/ml) (Eisen et al. 2008).

Ficolin-2 is expressed in the liver and serum concentration varies from 1340 to 14,700 ng/ml in adults, with a median of 3370 ng/ml (Sallenbach et al. 2011). The *+6424 G* > *T* variant in exon 8 of the *FCN2* gene leads to amino acid substitution within the firbrinogen-like domain of ficolin-2, and the mutation has a gene-dose effect on ficolin-2 serum levels (Munthe-Fog et al. 2007). The median serum levels of wild-type (*G/G*), heterozygotes (*G/T*) and homozygotes (*T/T*) have been shown to be 5100, 2200 and 900 ng/ml, respectively (Kilpatrick et al. 2013; Munthe-Fog et al. 2007). Thus, homozygosity does not lead to absolute ficolin-2 deficiency but is generally referred to as ficolin-2 insufficiency (Kilpatrick and Chalmers 2012). Concomitant with the variant’s downregulating effect is increased binding affinity for GlcNAc (Hummelshoj et al. 2005). Allele frequency has been reported to be between 0.12 and 0.14 in Caucasians (Damman et al. 2012; Herpers et al. 2006; Kilpatrick et al. 2013; Munthe-Fog et al. 2007; Ojurongbe et al. 2012). Low levels of ficolin-2 have been associated with prematurity, low birth weight and infections in neonates, childhood infections combined with allergic diseases and preeclampsia (Atkinson et al. 2004; Cedzynski et al. 2009; Swierzko et al. 2009; Wang et al. 2007). In addition, the *FCN2* + *6424* variant has been linked to the early onset of *Pseudomonas aeruginosa* in cystic fibrosis patients (Haerynck et al. 2012).

Ficolin-3 is the most abundant LP PRM in serum and the most potent LP activator in vitro (Hummelshoj et al. 2008). Its concentration varies tenfold (6100–60,300 ng/ml) in adults, with a median of 19,500 ng/ml (Sallenbach et al. 2011). The *+1637delC* variant in exon 5 of the *FCN3* gene is a frameshift mutation leading to the truncation of the C-terminal end of the ficolin-3 protein (Munthe-Fog et al. 2008). The variant has gene-dose-dependent effect on ficolin-3 serum levels, and homozygotes have no detectable ficolin-3 in serum (Michalski et al. 2011; Munthe-Fog et al. 2009; Munthe-Fog et al. 2008). Gene frequency has been reported to be rare (0.01–0.02) suggesting the crucial functions of ficolin-3 (Michalski et al. 2011; Munthe-Fog et al. 2009). Congenital ficolin-3 deficiency (+*1637delC* homozygotes) has so far only been confirmed in six individuals including two prematures, one infant and three adults. These subjects were affected by various morbidities (though nothing in common), such as invasive necrotizing eneterocolitis (NEC), perinatal *Streptococcus agalactiae* infections, congenital heart disease, recurrent severe pulmonary infections, brain abscesses, and severe nephrotic syndrome (Michalski et al. 2011; Michalski et al. 2015b; Munthe-Fog et al. 2009; Schlapbach et al. 2011). One of the homozygous adult was found in a healthy control group (Metzger et al. 2015).

MASP-2 serum levels ranges from 125 to 1150 ng/ml, with a median of 416 ng/ml (Sallenbach et al. 2011). The *p.D120G* mutation results in impaired binding to both MBL and ficolins (Stengaard-Pedersen et al. 2003; Thiel et al. 2009) and influences MASP-2 serum levels in a gene-dose-dependent manner. Homozygotes for the *p.D120G* variant have no detectable MASP-2 in serum and no MBL-mediated activation of complement (Stengaard-Pedersen et al. 2003). Allele frequency is 0.039 in Danish Caucasians (Thiel et al. 2007). Interestingly, the allele is not found in other populations such as Hong Kong Chinese, Zambian Africans and Amerindians in Brazil (Thiel et al. 2007). A total of thirteen *p.D120G* homozygotes have been published from case-control studies since the first case that was reported in the year 2003 (Stengaard-Pedersen et al. 2003). Six were unaffected or healthy (Garcia-Laorden et al. 2006; Olszowski et al. 2014; Segat et al. 2008; Sokolowska et al. 2015; Stover et al. 2005). Seven were detected in cohorts of recurrent respiratory infections, cystic fibrosis, colorectal cancer, hepatocellular carcinoma and pulmonary tuberculosis (Cedzynski et al. 2009; Cedzynski et al. 2004; Olesen et al. 2006; Segat et al. 2008; Sokolowska et al. 2015). Thus, the impact of MASP-2 deficiency (*p.D120G* homozygosity) in disease association is uncertain and it appears that homozygotes are found by chance. One might conclude from this that the LP is dispensible or redundant for human health because the current knowledge is that MASP-2 is crucial for LP complement activation. There are probably unidentified molecules and functions involved in the LP that could explain why MASP-2 deficiency is relatively common in apparently healthy individuals. In certain situations, being a *p.D120G* carrier may be relevant such as in the medical intensive care unit where it has been reported that the *p.D120G* allele was associated with increased risk of early death (Henckaerts et al. 2009).

Numerous studies have linked MBL deficiency with increased susceptibility to a variety of infectious and autoimmune diseases (reviewed in (Bjarnadottir and Ludviksson 2010; Dommett et al. 2006; Eisen and Minchinton 2003; Kilpatrick 2002; Thiel et al. 2006; Worthley et al. 2005). Several recent case-control studies have been investigating alternations in the distribution patterns of serum concentration of the early LP components in disease pathogenesis such as in patient groups with systemic lupus erythematosis (SLE), hemodialysis, pulmonary tuberculosis and acute liver failure (Chalmers et al. 2015; Ishii et al. 2011; Laursen et al. 2015; Troldborg et al. 2015a; Troldborg et al. 2015b). However, there are various factors that can influence serum levels, such as acute phase response and medication. Thus, genetic analysis may in certain settings provide more lines of reliable information. Studies involving determination on the combination of LP variants within LP genotypes remain scarce. To our knowledge, this is the first study that describes the frequency and distribution of the downregulating variants in the *FCN2*, *FCN3* and *MASP2* genes among *MBL2* genotypes in a healthy Caucasian population.

## Material and methods

### Subjects and samples

Between April and May 2010, EDTA blood was collected from a total of 498 volunteer blood donors from the Icelandic Blood Bank, and genomic DNA was isolated using high-salt procedure (Miller et al. 1988). The donors were Caucasians with age range of 18–60 years, and 125 were women and 375 were men. Informed consent was obtained from participants and only ethnic origin, gender and age was recorded. The study was approved by the Bioethics Committee of Iceland and the Data Protection Committee of Iceland.

### Genotyping

A real-time polymerase chain reaction (PCR) with subsequent melting curve (*T*_*m*_) analysis on amplicons was used to detect functional variants (rs1800450, rs1800451, rs5030737) in exon 1 and promoter (rs7096206) of the *MBL2* gene with the LightCycler Instrument (Roche Diagnostics) using a previously described method (Steffensen et al. 2003). *FCN2* genotyping was carried out using a sequence-specific primer PCR (SSP-PCR) to detect the functional variant (rs7851696) in exon 8 (Szala et al. 2013). Restriction fragment length polymorphism PCR (RFLP-PCR) was used to detect the base deletion *1637delC* (rs28357092) in the *FCN3* gene (Michalski et al. 2011). *MASP2* genotyping was carried out using SSP-PCR to detect the mutation (rs72550870) in the *MASP2* gene (Boldt et al. 2011).

### Statistical analysis

Allele frequency was compared between two and three genotype subgroups within the blood donor cohort using the chi-square test. When comparing more than three genotype subgroups, the Kruskal-Wallis H test was used followed by the Dunn’s multiple comparison post hoc test. The level of significance was set at 0.05 and the program package SPSS 11.0 (SPSS, Inc, Chicago, Ill) was used for processing the data.

## Results

### Frequency of *MBL2* genotypes and LP downregulating variants in the blood donor cohort

The frequencies of the structural *MBL2* variant alleles and *MBL2* genotypes are listed in Table 1. A total of 318 were wild-type *A*/*A* (63.9 %), 168 were *A*/*O* (33.7 %) and 12 were *O*/*O* (2.4 %). The *A*/*O* genotypes were genotyped for the downregulating *X* variant in the *MBL2* promoter. Forty-one of the *A*/*O* genotypes were found to harbour the *X* variant, i.e. 25 *XA*/*B*, 1 *XA*/*C* and 15 *XA*/*D*. Thus, 53 of the blood donors (10.6 %) were MBL deficient (*XA*/*O* and *O*/*O* genotypes). The calculated allele frequencies of the downregulating variants *FCN2* + *6424*, *FCN3* + *1637delC* and *MASP2 p.D120G* in the blood donors are shown in Table 2. Six individuals were homozygous for the *FCN2* + *6424* variant (*T*/*T*). In contrast, neither for the *FCN3* + *1637delC* variant nor the *MASP2 p.D120G* variant was detected in the healthy cohort.

**Table 1:** Distribution of *MBL2* genotypes and structural variant frequencies in the cohort

No. (%) of blood donors (*n* = 498)							Allele frequencies		
*A*/*A*	*A*/*B*	*A*/*C*	*A*/*D*	*B*/*B*	*B*/*D*	*D*/*C*	p*B*	p*C*	p*D*
318 (63.9)	114 (22.9)	8 (1.6)	46 (9.2)	7 (1.4)	4 (0.8)	1 (0.2)	0.13	0.01	0.05

**Table 2:** Frequency of the downregulating LP variants in the cohort

Variant	No. (%) wild types	No. (%) heterozygotes	No. (%) homozygotes	Allele frequency
*FCN2* + *6424 G* > *T*	405 (81) *G*/*G*	87 (17.5) *G*/*T*	6 (1.2) *T*/*T*	0.099
*FCN3* + *1637delC*	483 (97) *C*/*C*	15 (1.5) *-*/*C*	0 (0) *-*/*-*	0.015
*MASP2p.D120G A* > *G*	459 (92) *A*/*A*	39 (3.9) *A*/*G*	0 (0) *G*/*G*	0.039

### Distributon of the *FCN2* + *6424* variant among *MBL2*, *FCN3* and *MASP2* genotypes

**Fig. 1:**
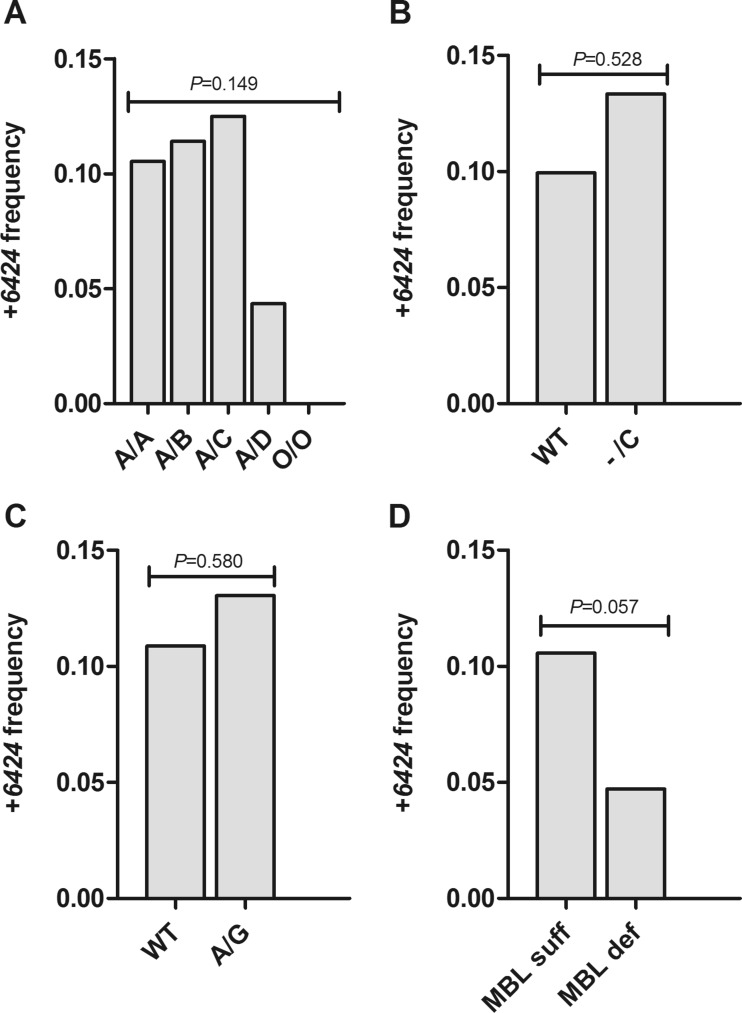
Frequency of the *FCN2* + *6424 G* > *T* variant among different genotypes in the blood donor cohort (*n* = 498). **a** Frequency among *MBL2* genotypes; *A*/*A* (*n* = 318), *A*/*B* (*n* = 114), *A*/*C* (*n* = 8), *A*/*D* (*n* = 46), *O*/*O* (*n* = 12). **b** Frequency between wild type (WT) (*n* = 483) and carriers of the *FCN3* + *1637delC* variant (*n* = 15). **c** Frequency between wild type (*n* = 459) and carriers of the *MASP2D120G* variant (*n* = 39). **d** Frequency between MBL-sufficient genotypes (*n* = 445) and MBL-deficient genotypes (*n* = 53). Allele frequency was compared with the Kruskal-Wallis test followed by Dunn’s multiple comparison pos hoc test (**a**) and the chi-square test (**b**–**d**)

A total of 93 subjects in the blood donor cohort carried the *FCN2* + *6424* variant and the calculated gene frequency was 0.099 (Table 2). Figure 1a demonstrates how the variant was distributed among the five known *MBL2* genotypes. Allele frequency did not significantly differ between the *MBL2* genotypes; however, the frequency was substantially low in *A*/*D* genotype carriers (0.043) and the variant was absent in *O*/*O* genotypes (Fig. 1a). The comparison of *FCN2* + *6424* frequency among *O*/*O* and *A*/*D* genotypes (*n* = 58) versus the *A*/*A*, *A*/*B* and *A*/*C* genotypes (*n* = 440) resulted in significant difference (0.034 versus 0.108, *P* = 0.013, chi-square test). Interestingly, the six *FCN2* + *6424* homozygotes (*T*/*T*) (Table 2) were only found among wild-type *MBL2* genotypes (*A*/*A*), whereas no homozygotes were found among individuals carrying the *MBL2* structural alleles (*A*/*O* and *O*/*O* genotypes). The *FCN2* + *6424* frequency did not differ between the heterozygous carriers of *FCN3* + *1637delC* versus wild-type (Fig. 1b) and the heterozygous carriers of *MASP2p.D120G* versus wild-type (Fig. 1c).

### The *FCN2* + *6424* variant in MBL deficiency

Previous studies indicate that ficolin-2 may be compensating for the lack of MBL (Ishii et al. 2011). Thus, we hypothesized that the *FCN2* + *6424* variant may be rare in MBL deficiency due to its downregulating effect on ficolin-2 levels (Kilpatrick et al. 2013; Munthe-Fog et al. 2007). The study cohort was divided into those carrying MBL-sufficient genotypes (*A*/*A* and *AY*/*O*) (*n* = 445) and those carrying MBL-deficient genotypes (*AX*/*O* and *O*/*O*) (*n* = 53) according to previous suggestions (Eisen et al. 2008). Subsequently, the *FCN2* + *6424G* > *T* allele frequency was determined in each group. The observed frequency of the variant tended to be lower in the MBL-deficient group than in the MBL-sufficient group (0.047 versus 0.0106, *P* = 0.054, chi-square test) (Fig. 1d).

### Distributon of the *FCN3* + *1637delC* variant among *MBL2*, *FCN2* and *MASP2* genotypes

**Fig. 2:**
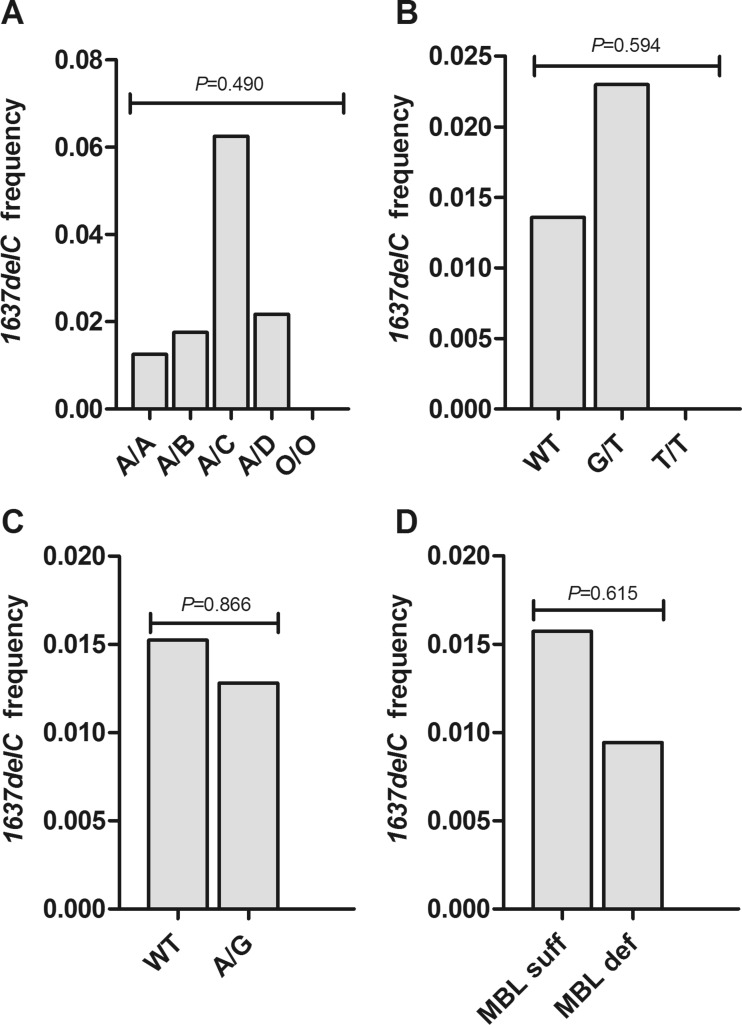
Frequency of the *FCN3* + *1637delC* variant among different genotypes in the blood donor cohort (*n* = 498). **a** Frequency among *MBL2* genotypes; *A*/*A* (*n* = 318), *A*/*B* (*n* = 114), *A*/*C* (*n* = 8), *A*/*D* (*n* = 46), *O*/*O* (*n* = 12). **b** Frequency among wild type (WT) (*n* = 483), heterozygous carriers of *FCN2* + *6424* (*G*/*T*) (*n* = 87) and homozygous carriers of *FCN2* + *6424* (*T*/*T*) (*n* = 6). **c** Frequency between wild type (*n* = 459) and carriers of the *MASP2D120G* variant (*A*/*G*) (*n* = 39). **d** Frequency between MBL-sufficient genotypes (*n* = 445) and MBL-deficient genotypes (*n* = 53). Allele frequency was compared with the Kruskal-Wallis test followed by Dunn’s multiple comparison pos hoc test (**a**) and the chi-square test (**b**–**d**)

There were 15 heterozygote carriers of the *FCN3* + *1637delC* in the blood donor cohort and calculated gene frequency was 0.015 (Table 2). The variant was equally distributed among the five known *MBL2* genotypes (Fig. 2a). The high frequency that was observed in the *A*/*C* genotype subgroup may be explained by the small size of the group (*n* = 8). The variant was absent in *O*/*O* genotype carriers. No significant deviations were found in *FCN3* + *1637delC* frequency between *FCN2* genotypes (Fig. 2b). Notably, four subjects were identified who were heterozygous for both downregulating variants in the ficolin genes, i.e. *FCN3* + *1637delC* (*-*/*C*) and *FCN2* + *6424* (*G*/*T*) (central bar in Fig. 2b). Thus, healthy individuals have intermediate serum levels of both ficolin-3 and ficolin-2. Interestingly, these four individuals were all high producers of MBL (two *AY*/*AY* genotypes, one *AY*/*B* genotype and one *AY*/*C* genotype). In contrast, neither *FCN3* + *1637delC* nor *FCN2* + *6424* were detected among *O*/*O* subjects which have undetectable MBL in serum. The *FCN3* + *1637delC* allele was equally distributed between wild-type and heterozygote *MASP2p.D120G* carriers (Fig. 2c).

### The *FCN3* + *1637delC* variant in MBL deficiency

We hypothesized that the *FCN3* + *1637delC* variant would be rare in MBL deficiency due to its downregulating effect on ficolin-3 levels (Munthe-Fog et al. 2009). One individual was heterozygous in the MBL-deficient group whereas 14 were found in the MBL-sufficient group (Fig. 2d). The only *FCN3* + *1637delC* carrier in the MBL-deficient group was an *AX*/*D* genotype carrier.

### Frequency of *MASP2p.D120G* among the *MBL2*, *FCN2* and *FCN3* genotypes

**Fig. 3:**
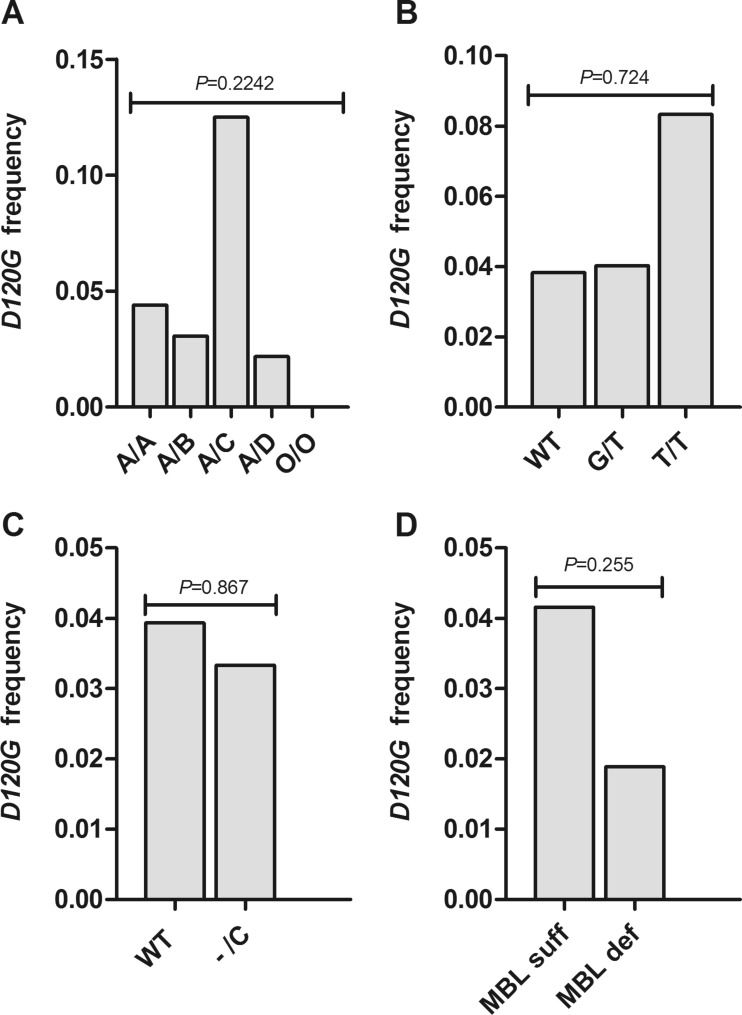
Frequency of the *MASP2D120G* variant among different genotypes in the blood donor cohort (*n* = 498). **a** Frequency among *MBL2* genotypes; *A*/*A* (*n* = 318), *A*/*B* (*n* = 114), *A*/*C* (*n* = 8), *A*/*D* (*n* = 46), *O*/*O* (*n* = 12). **b** Frequency among wild type (*n* = 483), heterozygous carriers of *FCN2* + *6424* (*G*/*T*) (*n* = 87) and homozygous carriers of *FCN2* + *6424* (*T*/*T*) (*n* = 6). **c** Frequency between wild type (*n* = 483) and carriers of the *FCN3* + *1637delC* variant (-/C) (*n* = 15). **d** Frequency between MBL-sufficient genotypes (*n* = 445) and MBL-deficient genotypes (*n* = 53). Allele frequency was compared with the Kruskal-Wallis test followed by Dunn’s multiple comparison pos hoc test (**a**) and the chi-square test (**b**–**d**)

We included the *MASP2p.D120G* variant in our analysis. It causes downregulation of MASP-2 in a gene-dose-dependent manner that is the same effect of the *FCN2* + *6424* and *FCN3* + *1637delC* variants. The MASP-2 protein is an enzyme activator of the LP and not one of the six recognition proteins of the LP. Thus, the compensation hypothesis would not be applied to this variant and one would not expect the frequency to differ. There were 39 *MASP2p.D120G* heterozygote carriers in the blood donor cohort and calculated gene frequency was 0.039 (Table 2). The observed gene frequency of *MASP2p.D120G* did not differ between *MBL2* genotypes, *FCN2* genotypes or *FCN3* genotypes (Fig. 3a–c). The elevated frequency in the *A*/*C* (*n* = 8) and *T*/*T* (*n* = 6) subgroups (0.143 and 0.083, respectively) may be due to the small number of individuals in these groups. The variant was absent in *O*/*O* genotype carriers (Fig. 2a). The frequency was low in MBL-deficient subjects but not statistically different from MBL-sufficient subjects (Fig. 3d).

## Discussion

The observed occurrence of *MBL2 A*/*A* and *A*/*O* genotypes in the blood donors is similar as has been observed among randomly selected individuals from a population of same ethnic origin (Dahl et al. 2004; Garred et al. 2006; Mead et al. 1997; Roy et al. 2002). However, only 2.4 % of the blood donors were of the *O*/*O* genotype whereas we have observed this ratio to be 5.6 % among randomly selected individuals from the Icelandic nation (*n* = 250, unpublished data). Similar findings have been observed in other studies. The ratio of *O*/*O* genotypes was 5–7 % when selected randomly (Dahl et al. 2004; Davies et al. 1995; Roy et al. 2002) but 2.5–2.8 % when selected for health (Kronborg et al. 2002; Lee et al. 2005). Furthermore, none of the *O*/*O* subjects in our healthy cohort were carriers of the downregulating variants (*FCN2* + *6424*, *FCN3* + *1637delC* or *p.D120G*). Thus, by selecting for health, one may expect to observe twofold fewer *O*/*O* genotypes than from a random selection and they may not be carriers of the downregulating LP variants. Therefore, being an *O*/*O* genotype carrier may be relevant when it comes to health and may be linked to morbidity, particularly when there is an additional LP component deficiency.

The observed gene frequency of the *FCN2* + *6424* variant (0.099) in the Icelandic Caucasian blood donors (*n* = 498) is slightly lower than has been observed for Dutch (*n* = 188) (0.136) and Danish Caucasian (*n* = 214) (0.118) blood donors and Dutch Caucasian (*n* = 1268) (0.137) kidney donors (Damman et al. 2012; Herpers et al. 2006; Munthe-Fog et al. 2007). Our results (from this study and unpublished) demonstrate that the downregulating *FCN2* + *6424* variant tends to be less frequent in MBL-deficient than in MBL-sufficient subjects. Supporting this observation are the findings of Ishii et al. who reported elevated levels of ficolin-2 among *O*/*O* genotypes (Ishii et al. 2011). Thus, ficolin-2 may be compensating for the lack of MBL. This conclusion is supported by the facts that ficolin-2 and MBL resemble in molecular structure, they are both produced in the liver, they both initiate the LP of complement via associated MASPs and they have been shown to bind a wide variety of pathogens some of which are in common (Degn and Thiel 2013; Kilpatrick and Chalmers 2012). It is conceivable that ficolin-2 is upregulated due to the lack of MBL, but we speculate, based on our results, that elevated ficolin-2 levels among *O*/*O* genotypes is due to the rarity of the downregulating *FCN2* + *6424* variant among MBL-deficient individuals. Thus, there may be a certain beneficial balance between MBL and ficolin-2 serum concentration for the host that has been maintained throughout evolution, and this may be controlled by the genetic variants.

The low gene frequency of *FCN3* + *1637delC* and the small sample size of the blood donor cohort preclude significant conclusions regarding the *FCN3* + *1637delC* gene frequency in MBL deficiency or low producers of ficolin-2 (*FCN2* + *6424* homozygotes). Therefore, further investigations in a cohort with a larger number of MBL-deficient genotypes and *FCN2* + *6424* homozygotes are warranted. The only *FCN3* + *1637delC* carrier found in the MBL-deficient group of our study cohort was of the *XA*/*D* genotype. Notably, the *D* variant is believed to have less dramatic effect on MBL levels than the *B* or *C* variants. The *D* variant chain is partly incorporated into stable higher oligomerized MBL explaining why MBL levels are generally higher among *A*/*D* genotypes than *A*/*B* and *A*/*C* genotypes (Garred et al. 2003). Furthermore, some investigators state that *D* allele carriers are intermediate MBL producers and should not be grouped as deficient (Degn et al. 2011). We have screened additional MBL-deficient adults (*n* = 140) for the *FCN3* + *1637delC* variant and found only one heterozygote carrier, and this individual was also of the *XA*/*D* genotype (unpublished data), further supporting our findings demonstrated here. Interestingly, homozygosity for *FCN3* + *1637delC* accompanied with the *AX*/*D* genotype was recently confirmed in an 11-month-old infant with congenital heart disease, pneumonia (without recurrence) and no severe infections after cardiac surgery (Michalski et al. 2015b). Thus, being a carrier of the *FCN3* + *1637delC* deletion and of the *AX*/*D* genotype does not seem to be life-threatening and not associated with severe recurrent infections.

Remarkably, the *FCN3* + *1637delC* variant was not found among subjects carrying genotypes *O*/*O*, *AX*/*B*, and *AX*/*C*. Thus, we speculate that having intermediate ficolin-3 levels or ficolin-3 deficiency accompanied with severe MBL deficiency (excluding the *D* variant, i.e. genotypes *O*/*O*, *AX*/*B* and *AX*/*C*) may have a substantial impact as co-morbid factor. In fact, the only individual that has been reported to be both *FCN3* + *1637delC* homozygous and of the *O*/*O* genotype was a premature baby (born at 35 weeks of gestation) with perinatal *Streptococcus agalactiae* infections, microcephaly, growth and mental retardation, and was diagnosed with attention deficit hyperactivity disorder (ADHD) at 3 years of age (Michalski et al. 2011). It has been suggested that ficolin-3 may be relevant in controlling commensal intestinal flora (Michalski et al. 2015a), and our previous studies show that *O*/*O* individuals are highly susceptible to gastrointestinal (GI) symptoms (Bjarnadottir et al. 2014). Thus, ficolin-3 and MBL may be relevant in the establishment of the gut microbiota at infancy. Recently, much attention has been on the association of the gut microbiota and microbiome with various disorders (Dave et al. 2012; Wang and Kasper 2014). Given the potential role of the LP in these complex interactions of the gut microbiota with its host and our findings reported here warrant further investigations on the impact of combined ficolin-3 and MBL deficiencies on microbiota and human health.

The observed frequency of the *p.D120G* allele in the Icelandic blood donor group is compatible with other studies including healthy Caucasians (Thiel et al. 2007). What we found noteworthy was that among the MBL deficiency genotypes, the variant was only associated with *AX*/*B* subjects and not with *AX*/*D* and *O*/*O* subjects. Ishii et al. reported significantly elevated levels of MASP-2 among *O*/*O* genotypes (Ishii et al. 2011). It is conceivable that MASP-2 was elevated in *O*/*O* genotypes because the downregulating variant *p.D120G* is absent. Furthermore, none of the 14 published *p.D120G* homozygotes were MBL deficient (personal communication) (Cedzynski et al. 2009; Cedzynski et al. 2004; Garcia-Laorden et al. 2008; Olesen et al. 2006; Sokolowska et al. 2015; Stengaard-Pedersen et al. 2003). Thus, we speculate that it may not be beneficial for the host to be a carrier of *p.D120G* and MBL deficiency (excluding the *AX*/*B* genotype). In contrast, the *p.D120G* was found equally distributed among sufficient (*G*/*G*), intermediate (*G*/*T*) and low producers of ficolin-2 (*T*/*T*).

Our results demonstrate how various LP deficiency variants are distributed among various LP genotypes in healthy individuals. In continuation, this data may become useful as a reference in disease association studies. Employing LP variant distribution pattern analysis in case-control studies may not replace determining the patterns of serum levels, but it may be an alternative and perhaps an innovative approach to investigate the impact of the LP on human health.
